# The Role of Blood Microbiome in the Development of Thyroid Cancer in Breast Cancer Survivors

**DOI:** 10.3390/cancers15184492

**Published:** 2023-09-09

**Authors:** Jeongshin An, Hyungju Kwon, Young Ju Kim

**Affiliations:** 1 Institute of Convergence Medicine Research, Ewha Womans University Mokdong Hospital, College of Medicine, Ewha Womans University, Seoul 07985, Republic of Korea; 2 Department of Surgery, Ewha Womans University Mokdong Hospital, College of Medicine, Ewha Womans University, Seoul 07985, Republic of Korea; 3 Department of Obstetrics and Gynecology, Ewha Medical Institute, College of Medicine, Ewha Womans University, Seoul 07804, Republic of Korea

**Keywords:** breast cancer, thyroid cancer, microbiome, *Bacillus*

## Abstract

**Simple Summary:**

Thyroid cancer often occurs as a second primary cancer in breast cancer survivors, but the cause remains unclear. Therefore, this study investigated a microbiome that could be related to the development of thyroid cancer in patients with breast cancer. *Bacillus* showed high concentrations in the thyroid cancer group and was associated with blood low-density lipoprotein (LDL) cholesterol and estrogen levels. In addition, *Bacillus* concentration increased with an increase in thyroid-stimulating hormone, which might lead to the development of thyroid cancer. Thus, thyroid cancer, which occurs as a secondary cancer in patients with breast cancer, was found to be associated with microbiomes, specifically *Bacillus*.

**Abstract:**

Patients diagnosed with breast cancer are likely to be diagnosed with thyroid cancer as a second primary cancer. Similarly, patients with thyroid cancer are likely to develop breast cancer. In this study, we found an association between these two types of cancers in the microbiomes of patients with breast cancer. Blood samples were collected from 96 patients with breast cancer, their bacterial extracellular vesicles were isolated, and their microbiomes were analyzed. After microbiome analysis, researchers performed thyroid function tests, estrogen levels, and thyroid ultrasound results of these patients, and the relationships among these parameters were analyzed. Based on the thyroid ultrasonography results, differences in the microbiome were confirmed in the normal, cyst, nodule, and thyroid lobectomy groups. We investigated the microbiome differences between normal thyroid and thyroid cancer. In particular, the abundance of the genus *Bacillus* is related to estrogen levels, which could affect thyroid abnormalities and increase thyroid-stimulating hormone levels. This study explains the causes of thyroid cancer in patients with breast cancer using microbiomes and serological tests for thyroid hormones and estrogen. These can be used as basic data for preventing thyroid cancer in patients with breast cancer.

## 1. Introduction

Breast cancer is the most common cancer in women worldwide [1], including in South Korea [2]. Many patients with breast cancer have thyroid cancer as their second primary cancer, and there are various hypotheses regarding this [3]. Hormonal factors, radiation exposure, and genetic problems are causes of thyroid cancer in patients with breast cancer [4]; and estrogen and thyroid receptors are particularly involved [5]. A microbiome related to estrogen metabolism, called the “estrobolome”, is involved in the development of breast cancer [6]. Hence, it is necessary to consider the role of microbiomes in the development of thyroid cancer in patients with breast cancer.

The blood microbiome maintains its health through a symbiotic balance with the host [7]. However, when this balance is disrupted, dysbiosis occurs, leading to diseases such as carcinosis [8]. For example, specific microbiomes in blood samples are associated with breast cancer diagnosis. In patients with breast cancer, the genera *Enterobacter*, *Bacteroides*, *Bifidobacterium*, *Fecalibacterium*, and *Subdoligranulum* are enriched, while the abundance of the genera *Pseudomonas*, *Streptococcus*, *Lactobacillus*, *Staphylococcus,* and *Acinetobacter* are decreased [9]. In particular, the microbes in the estrobolome vary between patients with breast cancer and healthy controls [6]. Microbes known to play a role in preventing breast cancer include the genera *Klebsiella* and *Staphylococcus* [6,10]. Therefore, it is possible to identify microbes involved in the development of thyroid cancer in patients with breast cancer.

When a patient with breast cancer is diagnosed with thyroid cancer as their second primary cancer, exposure to risk factors for thyroid cancer precedes the diagnosis. Representative risk factors and pathogenic hits for thyroid cancer include radiation exposure to the head and neck during adolescence, genetic factors such as *RET*, *BRAF*, and *RAS* gene mutations, and previous thyroid diseases [11,12]. This study focused on previous thyroid abnormalities. In the relationship between breast cancer and thyroid cancer, a significant association with benign thyroid disease was also found in a previous study [13]. Thyroid nodules are clinically important because thyroid cancer is diagnosed in approximately 4.0–6.5% of thyroid nodules [14]. Larger thyroid nodules and higher thyroid-stimulating hormone levels are associated with a higher risk of thyroid cancer [15,16]. Moreover, increased thyroid hormone receptor α and estrogen receptor α expression are also associated with the development of thyroid cancer [5].

In this study, risk factors for thyroid cancer, such as thyroid function tests, estrogen and low-density lipoprotein (LDL) cholesterol levels, and thyroid ultrasonography results, were analyzed along with the microbiome. Microbiome analysis revealed a microbe that acts in the process of carcinogenesis from breast to thyroid cancer. In addition, the role of this microbe was explained through its relationship with the serum test results for thyroid and estrogen.

## 2. Materials and Methods

### 2.1. Patient Characteristics

Blood samples from 96 patients with breast cancer were obtained from Ewha Womans University Mokdong Hospital (Table 1). Male patients and patients with genetic abnormalities such as BRCA 1 or 2 genes were excluded from this analysis. The mean age of the enrolled patients was 51 years, and all participants were diagnosed with stage 0–3 breast cancer. Patients had a mean BMI of 23.2 ± 3.5 kg/m^2^. All samples were obtained before surgery, radiotherapy, or chemotherapy. The microbial deoxyribonucleic acid (DNA) of the blood samples was analyzed. LDL cholesterol, high-density lipoprotein (HDL) cholesterol, triglyceride (TG), and total cholesterol levels were measured in the serum samples. Thyroid function test results were obtained within one month before and after blood sampling. Thyroid-stimulating hormone (TSH) and triiodothyronine (T3) were administered to all patients, whereas thyroxine (T4) and free T4 were administered to 64 and 58 patients, respectively. Of these, 37 underwent thyroid ultrasound during a follow-up period, which was approximately 5.9 years on average and 8 years maximum, after breast cancer surgery, and the results were classified into four categories. Patients with normal thyroid ultrasound findings were assigned to the normal group, and those with only cysts in the thyroid were assigned to the cystic group. If multiple thyroid nodules were found, the patients were divided into nodule groups. Patients with thyroid nodules underwent cytology only when deemed necessary. If a nodule is clearly benign, an ultrasound follow-up is typically performed instead of cytology as the standard management method for benign thyroid nodules [17]. Patients with thyroid lobectomy due to thyroid cancer (follicular carcinoma) or goiter were classified with the post-operative group. Patients who underwent thyroid lobectomy had part of their thyroid gland removed due to abnormalities. Although the remaining section of the thyroid might have been normal, this study hypothesized that their microbiome would differ from that of healthy individuals. This study was approved by the Institutional Review Board of the Ewha Womans University Hospital (IRB No. EUMC 2014-10-005).

### 2.2. DNA Extraction and Next-Generation Sequencing

All blood samples were collected in serum separator tubes and centrifuged at 3000 rpm for 15 min at 4 °C. The extracellular vesicles (EVs) were purified using a method described in previous studies [9,10]. EV DNA was purified from the serum using a DNeasy PowerSoil kit (QIAGEN, Hilden, Germany). EV DNA was quantified using a QIAxpert kit (QIAGEN). The DNA was amplified using the 16s_V3_F (5′-TCGTCGGCAGCGTCAGATGTGTATAAGAGACAGCCTACGGGNGGCWGCAG-3′) and 16s_V4_R (5′-GTCTCGTGGGCTCGGAGATGTGTATAAGAGACAGGACTACHVGGGTATCTAATCC-3′) primers [9,10]. Libraries and amplicons were prepared according to the Illumina manual and sequenced using a MiSeq platform (Illumina, San Diego, CA, USA).

### 2.3. Microbiome Analysis 

The profiling program MDx-Pro ver.2 (MD Healthcare, Seoul, Korea) was used for taxonomic assignments. To trim the primer sequences, Cutadapt (version 1.1.6) was used following which the sequences were merged with the Context-Aware Scheme for Paired-End Read (CASPER). Sequences less than 350 bp or greater than 550 bp in length with a Phred quality score of less than 20 were discarded to obtain high-quality sequencing reads. Operational taxonomic units were assigned at the genus level with a similarity threshold of 97% using the de novo VSEARCH clustering method. UCLUST and QIIME 1.9.1, based on the Silva 132 database, were used for taxonomy assignment at the species level. When the taxonomic information in the database was insufficient, we assigned the next highest taxonomic level. LEfSe analysis for functional biomarker discovery was performed using EzBioCloud based on PKSSU4.0 [18]. Protein interactions were analyzed using the STRING database (version 11.5). This study was conducted as a part of a larger project [9].

### 2.4. Statistical Analysis

Microbiome differences were identified using the Wilcoxon rank-sum test. Group clustering at the individual taxon level from phylum to species was performed using principal coordinate analysis (PCoA) based on the Bray–Curtis dissimilarity distance. Results were considered significant when *p* < 0.05. All analyses were performed using R version 3.6.1 and Prism version 9 software.

## 3. Results

### 3.1. Diversity

**Figure 1:**
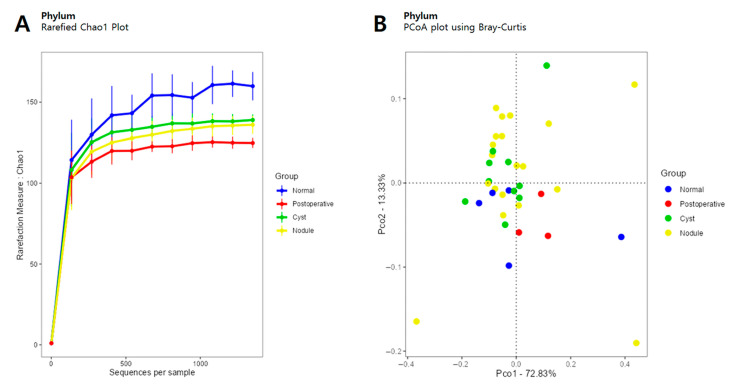
The difference in microbial diversity according to thyroid status in patients with breast cancer. (**A**) Alpha diversity determined using the Chao1 plot. (**B**) Beta diversity determined using principal coordinate analysis (PCoA) with the Bray–Curtis indices at the phylum level. Blue: normal thyroid group, red: post-operative group (thyroid lobectomy due to thyroid cancer or goiter), green: thyroid cyst group, yellow: thyroid nodule group.

The alpha and beta diversities were analyzed in breast cancer patients in the four groups according to their thyroid conditions (Figure 1). Alpha diversity was determined using the observed Chao1, Shannon, and Simpson methods (Supplementary Figure S1). Patients in the normal thyroid, post-operative, cyst, and nodule groups included 5, 3, 10, and 19 patients, respectively. Patients in the post-operative group included one patient with thyroid cancer and two with goiter. The normal and cyst groups showed similar mean values for observed richness, and the nodule and post-operative groups showed similar values of observed richness, lower than the normal group.

The Chao1 index was high in the normal group and low in other thyroid abnormalities (Figure 1A). Conversely, the Shannon index showed low values in normal thyroid and high values in thyroid abnormalities. For beta diversity, the PCoA plot was confirmed using the Bray–Curtis index, and the diversity from phylum to species was compared (Figure 1B). At the phylum level, the post-operative group was included in the normal group category and there was a tendency for the cyst and nodule groups to overlap; however, the difference was not statistically significant (*p* = 0.581).

**Figure 2:**
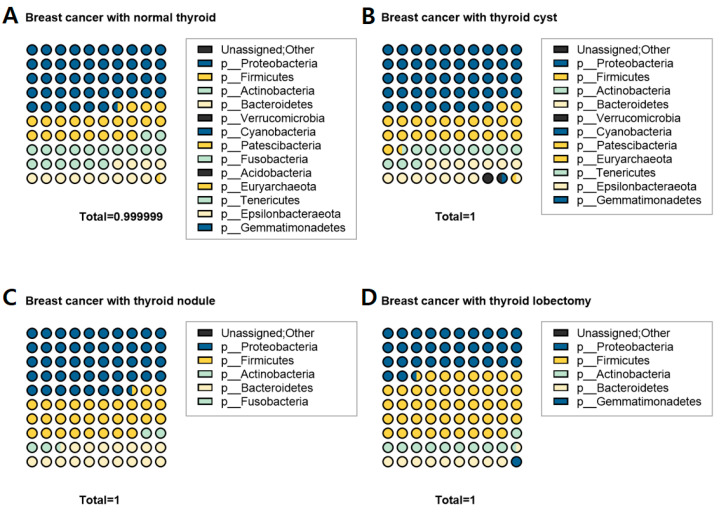
Metagenome profiles in the serum of patients with breast cancer according to thyroid status. (**A**) The composition of the microbiome at the phylum level in patients with breast cancer in the normal thyroid, (**B**) thyroid cyst, (**C**) thyroid nodule, and (**D**) post-operative (thyroid lobectomy) groups.

When comparing the four groups according to thyroid status, the phyla Proteobacteria, Firmicutes, Actinobacteria, and Bacteroidetes were common to all groups (Figure 2). The abundance of bacteria in the phyla Proteobacteria, Firmicutes, Actinobacteria, and Bacteroidetes were 31.8 ± 18.0%, 42 ± 20.6%, 12.8 ± 4.2%, and 11 ± 1.9%, respectively, in the normal thyroid group; 42.1 ± 5.9%, 28.9 ± 7.7%, 11.3 ± 3.8%, and 14.6 ± 3.3%, respectively, in the cyst group; and 35.7 ± 14.9%, 31.6 ± 14.1%, 13.0 ± 4.7%, and 15.4 ± 4.7%, respectively, in the nodule group. The normal thyroid group had more than 13 phyla (except those unassigned; Figure 2A). The cyst group had 11 phyla, and the nodule group had five (Figure 2B,C). The phyla Fusobacteria and Acidobacteria were absent in the cyst group compared to the normal thyroid group. The nodule group lacked the phyla Verrucomicrobia, Cyanobacteria, Patescibacteria, Acidobacteria, Euryarchaeota, Tenericutes, Epsilonbacteraeota, and Gemmatimonadetes (Figure 2C). The thyroid lobectomy (post-operative) group had five phyla, similar to the nodule group (Figure 2D). However, unlike the nodule group, the phylum Fusobacteria was absent, while Gemmatimonadetes were present in the post-operative group.

### 3.2. Microbiome Analysis According to Thyroid Ultrasonography

**Figure 3:**
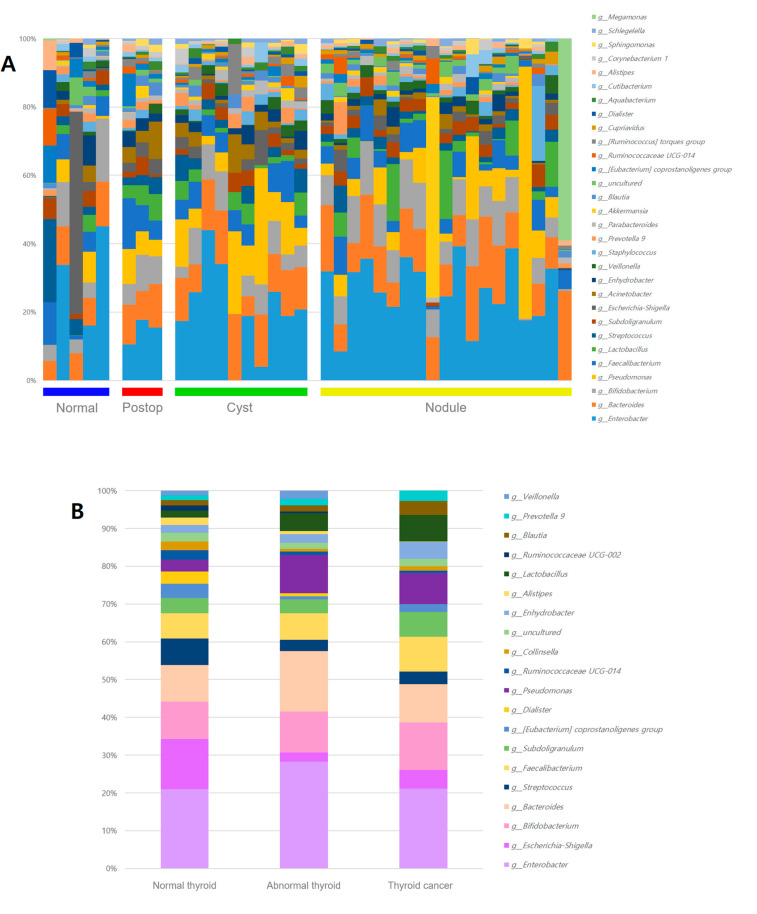

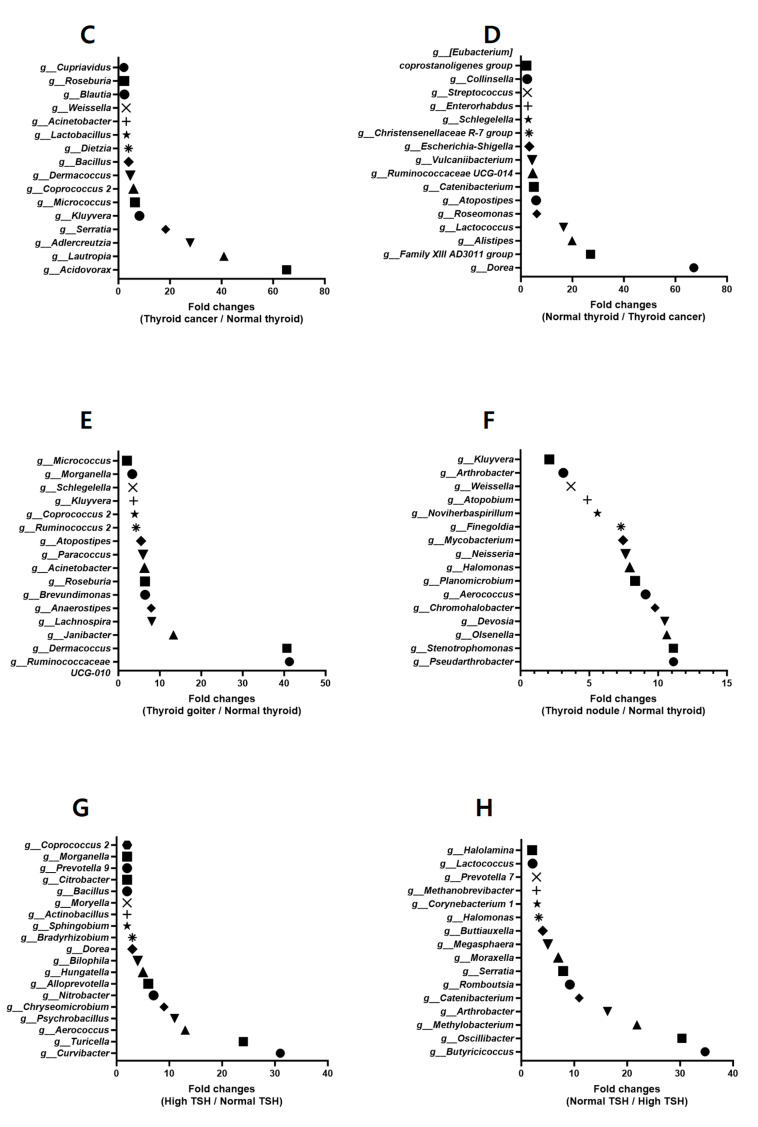
Metagenome profiles at the genus level in the serum of patients with breast cancer with various thyroid statuses (**A**) according to four groups consisting of normal: normal thyroid group, postop: post-operative (thyroid lobectomy) group, cyst: thyroid cyst group, and nodule: thyroid nodule group, and (**B**) three groups consisting of normal thyroid, abnormal thyroid (cysts and nodules), and thyroid cancer groups. Scatter plots showing fold changes of the abundant microbiome in (**C**) the thyroid cancer (above two folds) compared to the normal thyroid, (**D**) the normal thyroid (above two folds) compared to the thyroid cancer, (**E**) the thyroid goiter (above two folds) compared to the normal thyroid, (**F**) the thyroid nodule (above two folds) compared to the normal thyroid, (**G**) the high TSH compared the normal TSH, and (**H**) the normal TSH compared to high TSH group. TSH: thyroid-stimulating hormone.

**Figure 4:**
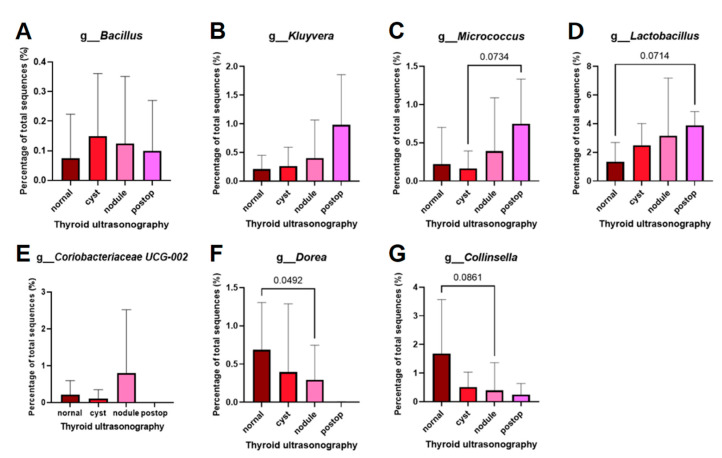
Bar graphs showing the genera that tended to differ in patients with breast cancer according to thyroid status. (**A**) *Bacillus*, (**B**) *Kluyvera*, (**C**) *Micrococcus*, (**D**) *Lactobacillus*, (**E**) *Coriobacteriaceae* UCG-002, (**F**) *Dorea*, and (**G**) *Collinsella*.

Patients with breast cancer were divided into four groups according to their thyroid status, and 30 microbiomes were selected at the genus level (Figure 3A). Similar to the comparison at the phylum level, patients with normal thyroids at the genus level had more types of microbiomes than patients with thyroid abnormalities. The difference between the thyroid cancer and the thyroid abnormality (cysts or nodules) group was compared by selecting the 20 genera in the order of the microbiome abundance based on the normal thyroid group (Figure 3B). The microbiomes of the thyroid cancer, which were more than two-fold higher than those of the normal thyroid group, are illustrated in Figure 3C. Figure 3D demonstrates the microbiomes of the normal thyroid group, which were more than two times higher than those found in the thyroid cancer group. The microbiomes specific to goiters or nodules, which were observed to be more than twice as abundant compared to those of the normal thyroid group, are illustrated in Figure 3E,F. According to the level of thyroid-stimulating hormone (TSH), the normal (0.3~4.1 µIU/mL) and high (above 4.2 µIU/mL) TSH groups were compared to each microbiome. Figure 3G lists the microbiome enriched in the high TSH group, while the microbiome enriched in the normal TSH group is shown in Figure 3H. Among these, microbiomes deficient in normal thyroid based on ultrasound results and abundant in thyroid abnormalities, and vice versa, were identified as targets at the genus level (Figure 4). The genera that showed a high tendency to cause thyroid abnormalities were *Bacillus*, *Kluyvera*, *Micrococcus*, *Lactobacillus*, and *Coriobacteriaceae* UCG-002 (Figure 4A–E). Conversely, the genera that tended to be enriched in the normal thyroid were *Dorea* (*p* = 0.0492) and *Collinsella* (*p* = 0.0861) (Figure 4F,G). Compared to the normal thyroid group, *Kluyvera, Bacillus,* and *Coriobacteriaceae UCG-002* increased by 2.7, 1.8, and 1.7 times, respectively, in the abnormal thyroid group. In the thyroid tumor group, *Bacillus* and *Kluyvera* showed a 4-fold and 8.1-fold increase, respectively, when compared to the normal thyroid group (Figure 3C).

### 3.3. Estrogen-Related Microbiome According to Microbiome Abundance

**Figure 5:**
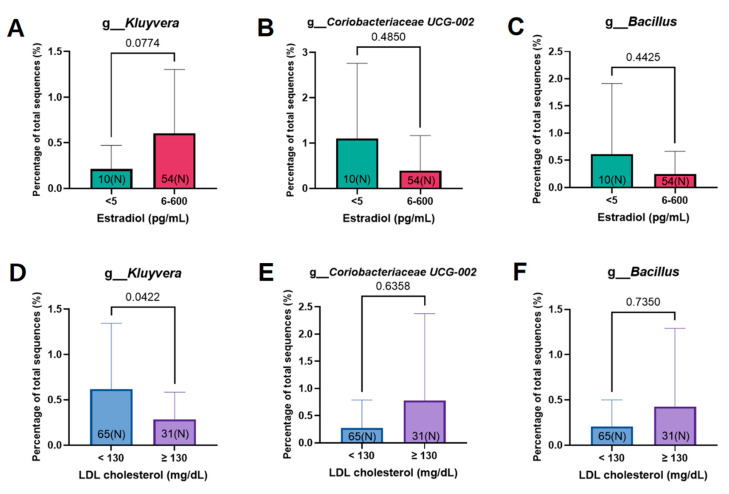
The genera that tended to differ in abundance depending on estrogen and LDL cholesterol levels. Shown are the differences in the percentage of the total sequence in the genera (**A**) *Kluyvera*, (**B**) *Coriobacteriaceae* UCG-002, and (**C**) *Bacillus* according to estradiol level. Also shown are the differences in the percentage of the total sequence in the genera (**D**) *Kluyvera*, (**E**) *Coriobacteriaceae* UCG-002, and (**F**) *Bacillus* according to LDL cholesterol level.

**Figure 6:**
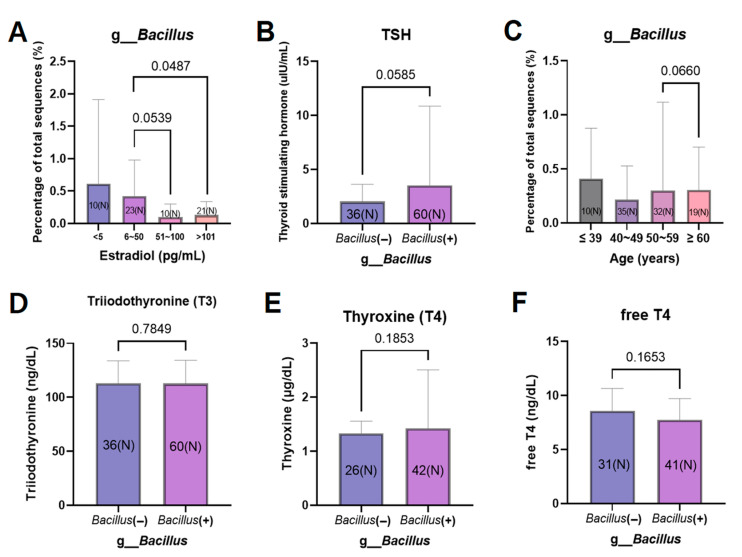
Relationship between genus *Bacillus* and thyroid function test results in patients with breast cancer. (**A**) Percentage of *Bacillus* sequences out of the total sequences according to estradiol level. (**B**) Thyroid-stimulating hormone levels with or without *Bacillus*. (**C**) Percentage of *Bacillus* sequences out of the total sequences according to age. Levels of (**D**) triiodothyronine, (**E**) thyroxine, and (**F**) free thyroxine with or without *Bacillus*.

Before identifying which microbiomes were related to blood estrogen levels, those related to LDL cholesterol levels were identified (Figure 5A–C). Since cholesterol is a precursor of estrogen, it was expected that LDL cholesterol and estradiol levels would be inversely proportional, and the result was as expected. Of the microbiomes with a tendency to be involved in thyroid abnormalities, *Kluyvera*, *Coriobacteriaceae* UCG-002, and *Bacillus* were found to be associated with LDL cholesterol (Figure 5A–C). *Kluyvera* was more prevalent in patients with normal LDL cholesterol (*p* = 0.0422), and their estradiol levels tended to be higher at 6–600 pg/mL compared to <5 pg/mL (*p* = 0.0774) (Figure 5A,D). *Coriobacteriaceae* UCG-002 and *Bacillus* showed an opposite pattern to *Kluyvera* (Figure 5B,C,E,F). *Coriobacteiraceae* UCG-002 and *Bacillus* were more prevalent in hyperlipidemic patients with LDL cholesterol levels ≥ 130 mg/dL, and in the group with estradiol levels < 5 pg/dL, but were not statistically significant. However, with respect to estradiol concentration, *Bacillus* decreased in a dose-dependent manner, unlike *Kluyvera* and *Coriobacteriaceae* UCG-002, which was statistically significant (*p* = 0.0487) (Figure 6A). In particular, the genus that showed a high tendency to cause thyroid cancer and high TSH was *Bacillus* (Figure 6B).

The abundance of the genera *Kluyvera* and *Coriobacteriaceae* UCG-002 did not increase or decrease with estradiol concentration. After analyzing the relationship between *Bacillus* and estradiol concentration, the relationship between thyroid function test and *Bacillus* was analyzed. The thyroid-stimulating hormone (TSH) level was 1.4 µIU/mL (*n* = 36) without the *Bacillus* microbiome and 2.1 µIU/mL (*n* = 60) with the *Bacillus* microbiome, showing a higher mean value in the presence of the *Bacillus* microbiome (*p* = 0.0585) (Figure 6B). Therefore, *Bacillus* abundance was associated with decreased estradiol and increased TSH. The relationship between *Bacillus* and menopause was also confirmed by checking *Bacillus* abundance according to age; however, no correlation was found between age and *Bacillus* abundance (Figure 6C). Triiodothyronine (T3), thyroxine (T4), and free T4 levels showed no significant difference in the presence or absence of *Bacillus* (Figure 6D–F).

**Figure 7:**
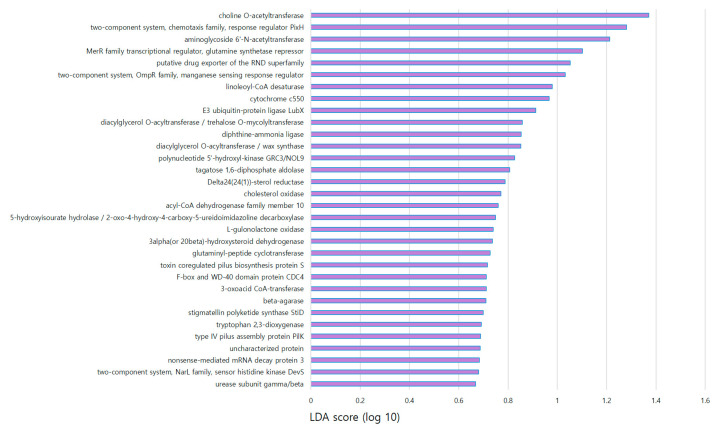
Functional biomarker discovery in patients with breast cancer in the thyroid nodule group compared to the normal thyroid group based on linear discriminant analysis (LDA) and effect size (LEfSe) analysis (*p* < 0.05).

**Figure 8:**
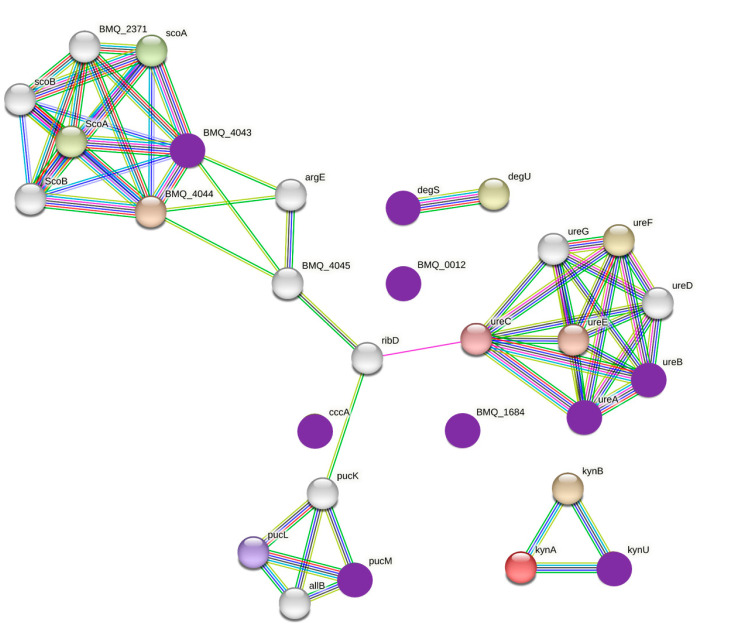
Comparison of protein interactions among functional biomarkers in patients with breast cancer in the thyroid nodule group compared to the normal thyroid group via the STRING database. The analysis was conducted with the bacterial database as the standard organism. The purple circles are proteins that match the specific bacteria such as *Bacillus amyloliquefaciens* in the database of functional biomarkers.

When comparing the normal and nodule groups and identifying the genes with *p* < 0.05 and similar functions, the difference was confirmed through the linear discriminant analysis (LDA) score (Figure 7). The most significantly differentially expressed gene in the nodule group was choline O-acetyltransferase (*p* = 0.016), followed by the two-component system, chemotaxis family, response regulator PixH (*p* = 0.016), and aminoglycoside 6’-N-acetyltransferase (*p* = 0.020). When comparing the number of sequences, diacylglycerol O-acyltransferase/wax synthase was 5.6-fold higher (*p* = 0.008), linoleoyl-CoA desaturase was 3.3-fold higher (*p* = 0.016), and cholesterol oxidase was 2.8-fold higher (*p* = 0.025) in the thyroid nodule group than the normal group. Functional markers related to protein interactions were analyzed based on the bacterial database using String (version 11.5). *Bacillus amyloliquefaciens* had the most matching proteins, and these are marked with purple circles in Figure 8. Protein interactions were divided largely into three groups, Acetyl-CoA C-Acyltransferase-related proteins, ureases, and the 5-hydroxyisourate hydrolase subfamily, respectively. Among them, Acetyl-CoA C-Acyltransferase is an enzyme that catalyzes the final step of fatty acid oxidation.

## 4. Discussion

Accompanying taxonomic profiling in this study, serological analysis suggested that microbiome influences the development of thyroid cancer in patients with breast cancer. To date, microbiome research has been limited to specific cancers, such as breast or thyroid cancer. For example, *Methylobacterium radiotolerans* is abundant in the microbiome of breast cancer tissue, while the genera *Enterobacter*, *Bacteroides*, and *Bifidobacterium* are abundant in the blood microbiome of patients with breast cancer [9,19]. *Streptococcus* and *Porphyromonas* are abundant in the gut microbiome of patients with thyroid cancer, and these genera are also enriched in patients with thyroid nodules compared to healthy controls [20]. Saliva microbiome analysis showed that genus *Acinetobacter* was more enriched in patients with thyroid cancer than healthy controls [21].

In this study, we focused on the fact that patients with breast cancer often develop thyroid cancer and that the microbiome was the cause of thyroid cancer in patients with breast cancer. Although some studies have suggested that breast and thyroid cancers are clinically related to estrogen, a direct cause remains unknown [22]. Instead, estrogen influences cellular functions by binding to nuclear receptors identified as estrogen receptors alpha and beta (ERα and Erβ, respectively) [23]. The expression of ERs, particularly ERα is involved in the development of thyroid cancer in patients with breast cancer [5]. In this study, patients with breast cancer were divided into four groups according to thyroid abnormalities. Microbiomes related to breast and thyroid cancers were analyzed (Figure 1 and Figure 2), and a specific microbiome was found that included, for example, *Bacillus*. It is worth noting that this microbiome is associated with the thyroid cancer group (Figure 3C).

The microbiome involved in estrogen metabolism is also involved in breast cancer. This “estrobolome” showed a significantly higher abundance in patients with breast cancer than in healthy controls [6]. Similarly, the microbiome related to thyroid cancer may also be present in patients with breast cancer, and this microbiome may be associated with cholesterol and estrogen metabolism. When analyzing the data used in this study, estrogen and LDL cholesterol showed a tendency for a negative correlation; however, no statistical significance was observed due to the small sample size (*p* = 0.974). Previous studies have shown that estrogen lowers LDL cholesterol [24]. Clinical trials have demonstrated that LDL cholesterol levels are reduced with estrogen replacement therapy (administered orally or subcutaneously). Moreover, estrogen replacement therapy is associated with LDL receptor activation [25]. In this study, the number of patients with thyroid cancer was just one; therefore, the subtype of breast cancer in patients with thyroid cancer could not be statistically indicated. However, the patient with thyroid cancer was a luminal A subtype related to the ER. Meaningful data can be obtained on breast cancer subtypes if more patient data are collected. According to previous studies, a higher genetic susceptibility to breast cancer corresponds with an increased risk of thyroid cancer overall; however, this association was not observed in triple-negative breast cancer [26]. While breast cancer is specifically related to the ER-positive subtype, thyroid cancer is associated with the overall types of thyroid cancer [26].

When identifying microbial candidates associated with LDL cholesterol and estradiol levels, the abundances of the genera *Kluyvera*, *Coriobacteriaceae* UCG-002, and *Bacillus* tended to be associated with serum levels of these molecules (Figure 5). These candidates have a high propensity for thyroid abnormalities or cancer in patients with breast cancer. *Kluyvera* is associated with energy or estrogen metabolism as it is abundant in patients with high estrogen and low LDL cholesterol levels. *Kluyvera* strains are known to ferment sugars and polyhydroxy alcohols [27]. Sugar fermentation in *Kluyvera* will make the sugar less likely to be stored as LDL cholesterol. This explains why *Kluyvera* was more prevalent in the LDL cholesterol < 130mg/dL group (low LDL cholesterol group) (Figure 5D). *Coriobacteriaceae* UCG-002 and *Bacillus* tended to be associated with low estradiol and high LDL cholesterol levels, although the results were not statistically significant (Figure 5). However, the abundance of *Bacillus* was inversely proportional to estradiol levels in a dose-dependent manner (Figure 6A). This suggests a correlation between *Bacillus* abundance and estrogen levels.

In the LEfSe results for identifying a functional biomarker specific to patients with breast cancer and thyroid nodules, the microbiome related to bacterial survival occupied the upper part of the LDA score, and factors related to fat metabolism were more abundant than in the normal thyroid group (Figure 7). In patients with breast cancer and thyroid nodules, the characteristic lipid metabolism-related factors included diacylglycerol O-acyltransferase, linoleoyl-CoA desaturase, and cholesterol oxidase. Diacylglycerol O-acyltransferase is a transmembrane protein and a key enzyme in lipid metabolism [28]. Linoleoyl-CoA desaturase converts fatty acids into different types, from α-linolenic acid (ALA) to eicosapentaenoic acid (EPA) [29]. Cholesterol oxidase is a bacterial flavoenzyme that catalyzes the oxidation of cholesterol to cholestenone [30]. The activities of these proteins in the nodule group may be associated with obesity and other metabolic diseases. Previous studies have investigated the relationship between thyroid nodules and metabolic diseases [31], which our microbiome results support. While matching functional biomarkers belonging to *Bacillus* spp. in the protein database, the protein belonging to the group related to Acetyl-CoA C-Acyltransferase was identified (Figure 8). Since the role of this protein is as an enzyme that catalyzes the final step of fatty acid oxidation [32], this biomarker has a possibility of being related to thyroid abnormalities with fat metabolism.

According to previous studies, the characteristics of *Bacillus* spp. are related to the degradation of estradiol into estrone. *Bacillus* spp. decompose the hydroxyl group of estradiol into alcohol to produce estrone [33]. Since decreased estradiol and increased TSH levels may be associated with postmenopausal women, we analyzed the association between *Bacillus* and age. We did not find a linear relationship between *Bacillus* abundance and age; therefore, the abundance of *Bacillus* was not related to age. Hyperlipidemia and hypothyroidism are related [34], and hypothyroidism is accompanied by increased TSH levels. However, it is difficult to define the relationship between hyperlipidemia and TSH directly; the presence of *Bacillus* could explain this niche. This study found that *Bacillus* spp. is involved in a series of processes that decrease estradiol levels and increase LDL cholesterol and TSH levels, which are risk factors for thyroid cancer. In this study, the correlation between Thyroid US and TSH was not analyzed. Since the specified *Bacillus* species, *Bacillus amyloliquefaciens,* was identified simply as the result of database matching, it cannot be specified as a species related to thyroid cancer (Figure 8). Another limitation is that not all patients performed the thyroid ultrasonography due to cost implications. However, this conclusion has not been reached before and could be one of the clues for preventing or treating thyroid cancer in patients with breast cancer in the future.

This study suggests the possibility that elevated TSH levels are involved in the pathogenesis of thyroid cancer in patients with breast cancer via the microbiome, such as the genus *Bacillus*. Estrogen and fat metabolisms are believed to be involved in the process.

## 5. Conclusions

Microbiome related to estrogen and fat metabolism is involved in developing secondary thyroid cancer in patients with breast cancer. *Kluyvera, Coriobacteriaceae UCG 002*, and *Bacillus* were increased in the group with thyroid abnormality in patients with breast cancer and were commonly related to estrogen and LDL cholesterol. TSH elevation was associated with the presence of *Bacillus*, which seems to be related to thyroid cancer and can be a development indicator of thyroid cancer as a second primary cancer in patients with breast cancer.

## Figures and Tables

**Table 1:** Patient characteristics.

Characteristics	Total
Female *n* (%)	96 (100%)
Age (year) ± SD	51.5 ± 11.1
Stage of breast cancer *n* (%)	
0	3 (3.1%)
I	44 (45.8%)
II	36 (37.5%)
III	13 (13.5%)
BMI (kg/m^2^)	23.2 ± 3.5
LDL cholesterol (mg/dL)	50.9 ± 13.2 (94.8%)
Estradiol (pg/mL)	109.1 ± 143.4 (66.7%)
Thyroid function tests	
TSH (µIU/mL) ± SD	3.0 ± 5.9 (100%)
T3 (ng/dL) ± SD	112.8 ± 21.2 (100%)
T4 (μg/dL) ± SD	8.1 ± 2.0 (70.8%)
Free T4 (ng/dL) ± SD	1.4 ± 0.9 (75%)
Thyroid US *n* (%)	
Normal	5 (5.2%)
Cyst	10 (10.5%)
Nodule	19 (20%)
Post-operative	3 (3.1%)
(thyroid lobectomy)	

## Data Availability

The unprocessed sequence data and the analyzed data from the metagenome analysis can be accessed via the Sequence Read Archive under the BioProject IDs: PRJNA834582.

## Supplementary Materials

The following supporting information can be downloaded at: https://www.mdpi.com/article/10.3390/cancers15184492/s1, Figure S1: Difference in microbial diversity according to thyroid status in patients with breast cancer.
