# Supplementary figures and images for: The Role of Blood Microbiome in the Development of Thyroid Cancer in Breast Cancer Survivors
Source: Cancers (Basel). 2023 Sep 9;15(18):4492. doi: 10.3390/cancers15184492 (PMC10526815; doi:10.3390/cancers15184492)

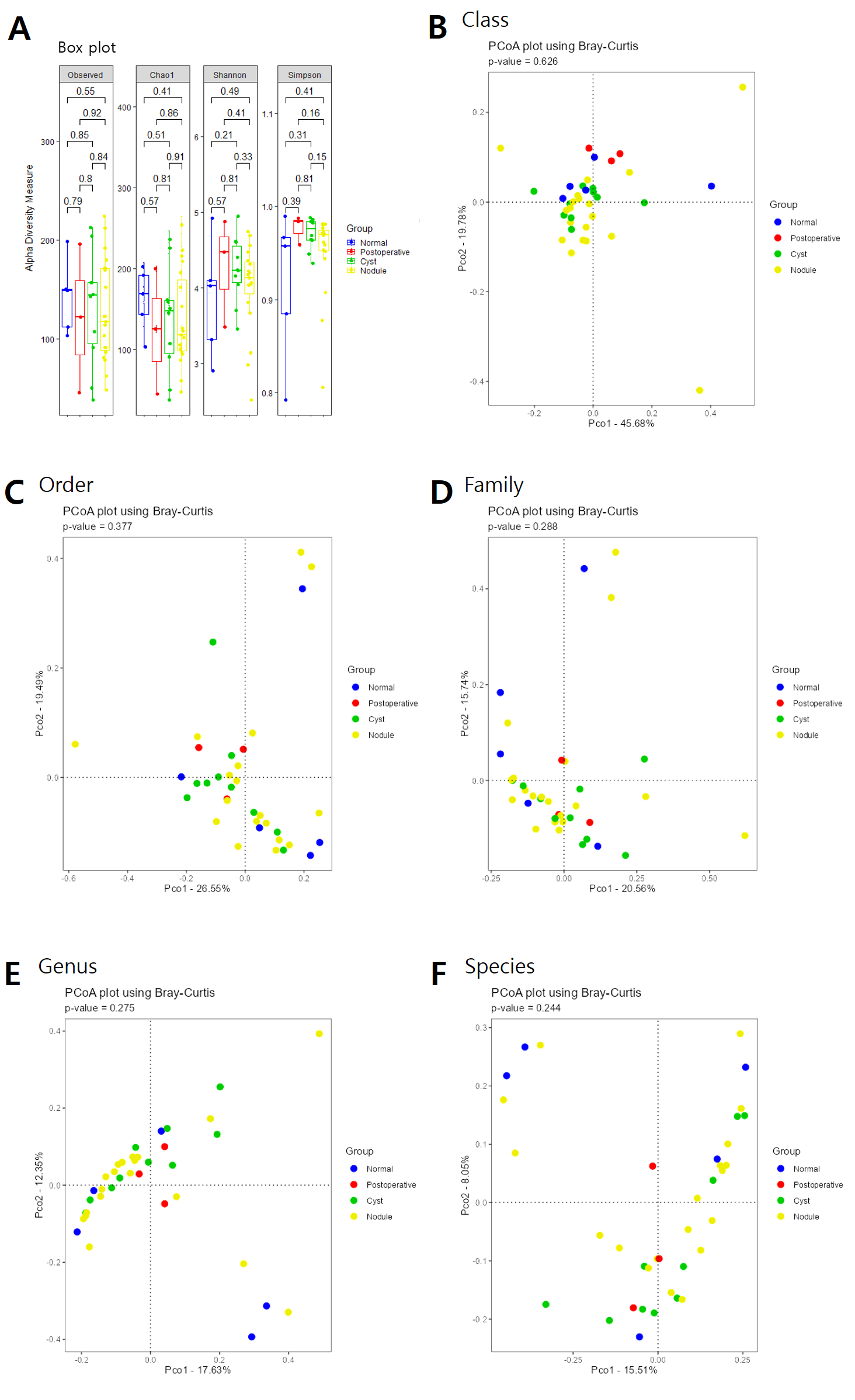

Supplement: Supplementary file 1 [file cancers-15-04492-s001.zip › cancers-2549680-supplementary.tif]
